# Modelling human protein interaction networks as metric spaces has potential in disease research and drug target discovery

**DOI:** 10.1186/1752-0509-8-68

**Published:** 2014-06-14

**Authors:** Emad Fadhal, Eric C Mwambene, Junaid Gamieldien

**Affiliations:** 1 South African National Bioinformatics Institute/ MRC Unit for Bioinformatics Capacity Development, University of the Western Cape, Bellville 7530, South Africa; 2 Department of Mathematics and Applied Mathematics, University of the Western Cape, Bellville 7530, South Africa

**Keywords:** Protein interaction networks, Drug discovery, Metric spaces, Core-periphery structure, Topological centrality, Essential proteins, Disease genes

## Abstract

**Background:**

We have recently shown by formally modelling human protein interaction networks (PINs) as metric spaces and classified proteins into zones based on their distance from the topological centre that hub proteins are primarily centrally located. We also showed that zones closest to the network centre are enriched for critically important proteins and are also functionally very specialised for specific ‘house keeping’ functions. We proposed that proteins closest to the network centre may present good therapeutic targets. Here, we present multiple pieces of novel functional evidence that provides strong support for this hypothesis.

**Results:**

We found that the human PINs has a highly connected signalling core, with the majority of proteins involved in signalling located in the two zones closest to the topological centre. The majority of essential, disease related, tumour suppressor, oncogenic and approved drug target proteins were found to be centrally located. Similarly, the majority of proteins consistently expressed in 13 types of cancer are also predominantly located in zones closest to the centre. Proteins from zones 1 and 2 were also found to comprise the majority of proteins in key KEGG pathways such as MAPK-signalling, the cell cycle, apoptosis and also pathways in cancer, with very similar patterns seen in pathways that lead to cancers such as melanoma and glioma, and non-neoplastic diseases such as measles, inflammatory bowel disease and Alzheimer’s disease.

**Conclusions:**

Based on the diversity of evidence uncovered, we propose that when considered holistically, proteins located centrally in the human PINs that also have similar functions to existing drug targets are good candidate targets for novel therapeutics. Similarly, since disease pathways are dominated by centrally located proteins, candidates shortlisted in genome scale disease studies can be further prioritized and contextualised based on whether they occupy central positions in the human PINs.

## Background

In order to develop an understanding of the roles of proteins in cellular dynamics, especially for the purposes of uncovering key players in disease development and for discovery of novel therapeutic targets, their physiological interactions must first be understood [1]. The specialized functions of the differentiated cell types which are assembled into tissues or organs depend on the combinatorial arrangements of proteins and their physical interactions. [2-4]. A major current challenge is therefore to understand the functions of various types of biological networks, including PINs. [5-16].

A predominant question in the analysis of PINs continues to be whether biological characteristics and functions of proteins such as lethality, physiological malfunctions and malignancy are intimately linked to the topological role proteins play in the network [17-21]. Much of the recent efforts in the analysis of protein-protein interaction (PPI) networks has therefore focused on finding functional dependencies between so-called hubs, defined as proteins involved in many interactions, and their topological roles in the network [22-24].

In the literature, nodes of PINs have been ranked by metrics such as degree, betweenness, eccentricity and closeness. The latter, which is defined as the reciprocal of the average geodetic distance between a given node and others, has particularly featured [25]. Using these metrics, a classification of proteins into core and periphery classes has been defined as a way to predict a protein’s relative importance in the network.

It has been reported that disease lethal genes are located in the ‘core’ of PPI networks [26-28]. Moreover, it has also been shown that highly connected proteins that are also functionally important are also *topologically* centered and are locally and globally important nodes in the core network [29] and that those with housekeeping functions are mainly located in close proximity to the topological core of the interactome [2]. Similarly, Vinogradov [30] showed that PINs of *Saccharomyces cerevisiae* and *Escherichia coli* consist of two large-scale modularity layers: central and peripheral, separated by a zone of depressed modularity. The categories of the central layer are mostly related to nuclear information processing, regulation and cell cycle, whereas the peripheral layer is dealing with various metabolic and energetic processes, transport and cell communication. Ignacio et al. [25] has developed ‘k-core decomposition’, a tool which enables the disentangling of the hierarchical structure of networks by progressively focusing on their central cores. The k-core analyses of PINs showed that drug-targets tend to be closer to the topological core [31].

We have recently showed, by using a more precise approach that formally models PINs as metric spaces and classifies proteins into zones based on their distance from the topological centre, that hub proteins are not distributed randomly and are in fact the main feature of zones closest to the network centre. [32]. Furthermore, we intimated that these zones have likely functional significance and proposed that centrally located proteins of both human functional protein interaction network (HFPIN) and the a curated human signalling network (HSN) may present good therapeutic targets. Here, we present further evidence to support our hypothesis and show that the functional and graph-theoretic properties of zones of both human PINs have biological significance. We provide a strategy of identifying possible potential for supporting therapeutic target discovery.

## Results and discussion

### Human PINs have a core-periphery structure when modelled as metric spaces

We modelled the HFPIN [33], which consists of 9448 nodes and 181706 interactions and the highly curated and currently largest available HSN [34,35], which consists of 6291 nodes and 62737 interactions (http://www.bri.nrc.ca/wang/). To do so, we first identified each network’s topological centre(s) using a formal method that finds the protein(s) that has the smallest maximal distance to other proteins in the network. This locates the protein at the *true* centre of the network, and does not assume that it has the highest number of connections/interactions. Once each network centre was identified, all proteins were categorized into zones based on their distance from the centre, which was defined as the *smallest* number of interactions that have to be traversed from any protein to reach the centre. For example, proteins were categorized as being in zone 1 if they directly interact with the centre and are this one distance unit away from it. Similarly, zone 2 proteins interact with *at least* one zone 1 protein and possibly also other proteins in other zones, but not directly with the centre and are thus 2 distance units away from it, etc.

The main aim of finding the centre of the network was to classify proteins into zones so as to further analyse them from a systems point of view. When we partitioned proteins into zones based on their distance from the centre, the metric structures of the networks could be summarized as follows: 

1. Both have a single protein as their topological centre: MAPK14 and MAPK1 respectively, for HFPIN and HSN. These proteins are members of the MAP kinase family and have been featured as drug targets [36,37].

2. HFPIN was found to contain 9 zones and HSN 6. We identified several features that support the core-periphery structure proposed for PPI networks. For purposes of further discussion, the zone closest to the centre will be referred to as zone 1.

In both networks, proteins in zone 1 was found to be the most connected with an average degree 86 and 67 respectively for HFPIN and HSN. Overall, 92% and 95% respectively of nodes are located in zones 1 to zone 3 for HFPIN and HSN. Zones 6 to 9 for HFPIN and zones 5 to 6 for HSN consist only of ‘quills’ (nodes that have degree 1) [32]. It is clear that the HPFIN and HSN structures therefore both have densely connected kernels that rapidly become more sparsely connected towards their peripheries (Table 1).

**Table 1:** Graph metrics of human PINs modeled with respect to distance from the centre

**PIN**	**Nodes**	**Edges**	**Diameter**	**Centre**	**Zones around centre**									
					**1**	**2**	**3**	**4**	**5**	**6**	**7**	**8**	**9**	
					374	4610	3464	578	104	14	2	1	1	Nodes
					**86**	**32**	**52**	2	2	1	1	2	1	Ave degree
HFPIN	9448	181706	13	**MAPK14**	3	1	1	1	1	1	1	2	1	Min degree
					531	430	393	14	6	2	2	2	1	Max degree
					0	173	653	307	56	12	1	0	1	# quills
					431	3527	1929	206	38	4				Nodes
					**67**	**24**	**7**	2	2	3				Ave degree
HSN	6291	62737	11	**MAPK1**	1	1	1	1	1	1				Min degree
					451	362	89	11	9	5				Max degree
					4	404	757	133	20	2				# quills
					542	6011	3352	367	61	4	1	1	1	Nodes
					**95**	**34**	**49**	2	2	1	2	2	1	Ave degree
CN	10573	210689	13	**MAPK3**	1	1	1	1	1	1	2	2	1	Min degree
					590	431	394	12	6	2	2	2	1	Max degree
					1	339	831	212	40	3	0	0	1	# quills

### Zones of both human PINs are functionally specialised

A summary of enriched pathway analysis reveals that specialization in protein functions and organizing principles are essentially identical in the two networks. While the total numbers of proteins in the two networks are significantly different, the proportions of proteins contributing to key functions and pathways in each zone are remarkably similar (Tables 2 and 3). Further, all between-zone differences in proportions of proteins involved in enriched functions were found to be statistically significant using a z-test (P < 0.01) in both networks. The distribution of all important cellular functions across the zones have essentially identical patterns.

**Table 2:** Functional specialization of HFPIN zones defined by distance from the network centre

**Enriched Pathway**	**Zone 1**	**Zone 2**	**Zone 3**	**Zone 4**	**Zone 5**
Signal transduction	38.1*%*	26.4*%*	-	-	-
Immune system	31.3*%*	8*%*	5.1*%*	-	-
Hemostasis	18.4*%*	5.9*%*	2.5*%*	-	-
Disease	17.5*%*	9.1*%*	4.4*%*	-	-
Gene Expression	7.9*%*	8.8*%*	11.9*%*	-	-
Metabolism	10.7*%*	9.1*%*	8.7*%*	11.3*%*	16.3*%*
Transmembrane transport of small molecules	-	2*%*	2.5*%*	3.9*%*	13.4*%*
Metabolic pathways	2.4*%*	4.8*%*	6.7*%*	12.7*%*	25*%*

Percentages indicate proportion of proteins in a zone belonging to the specific functional class.

**Table 3:** Functional specialization of HSN zones defined by distance from the network centre

**Enriched Pathway**	**Zone 1**	**Zone 2**	**Zone 3**	**Zone 4**
Signal transduction	40.6*%*	21.1*%*	9.3*%*	16*%*
Immune system	33.6*%*	16.2*%*	8.4*%*	-
MAPK signalling	28.9*%*	3.5*%*	-	-
Pathways in cancer	21.4*%*	5.7*%*	-	-
Disease	21*%*	10*%*	7.2*%*	9.8*%*
Hemostasis	16.5*%*	6.9*%*	3.3*%*	-
Cell Cycle	5.3*%*	7.6*%*	3.4*%*	-
Gene expression	7.2*%*	7.5*%*	8.7*%*	-
Metabolism of proteins	-	4.6*%*	4.6*%*	-

Percentages indicate proportion of proteins in a zone belonging to the specific functional class.

We observed statistically significant (Bonferroni corrected P-value < 0.01) functional enrichment in specific zones of the human PINs. Further, we observed in general that zones proximal to the centre appear to be more involved and specialized for key biological functions, with the proteins in those zones involved in only a few pathways. In contrast, zones distal from the centre appear to be more functionally diverse and are enriched for pathways involved in more routine functions. All differences between zones were confirmed to be statistically significant (P < 0.01). Zone 1 is highly enriched for proteins involved in signal transduction, the immune system, hemostasis and disease pathways and appears to constitute of a core of highly important interactions required for organism and cellular sensing and response to adverse environmental, biological and mechanical stresses. Zone 2 is also enriched for proteins involved in signal transduction and immune system pathways and is moderately enriched for gene expression and metabolic pathways, which are the main functional themes in zone 3. From zone 4 onwards, proteins have significantly less enrichment than zones closer to the centre, with metabolism, metabolic pathways, metabolism of proteins, membrane trafficking and transmembrane transport of small molecules being the main functional themes.

### The human functional protein interaction network has a highly connected signalling core

Due to the proportional statistical over-representation of signal transduction pathways in the zones closest to the centre, their known importance in cellular functions and their prominence as a drug target category, we explored the distribution of proteins having any signalling function, as well as functions related to regulation of signalling (Table 4). All differences between proportions of signalling related proteins between zones were found to be statistically significant (P < 0.01).

**Table 4:** Summary of cellular function in the central zones of HFPIN

**Cellular**	**# of proteins**	**Zone 1**	**Zone 2**	**Zone 3**
**function**				
Signalling pathway	3186	285 (76.2%)	2141 (46.4%)	760 (20.8%)
Positive signals	544	75 (20%)	386 (8.3%)	83 (2.2%)
Negative signals	449	57 (15.2%)	321 (6.9%)	71 (1.9%)
MAPK signalling cascade	394	93 (24.8%)	258 (5.5%)	43 (1.1%)
Apoptosis signalling pathway	38	13 (3.4%)	19 (0.4%)	6 (0.1%)
Positive regulation of apoptosis signalling	22	6 (1.6%)	13 (0.2%)	3 (0.1%)
Negative regulation of apoptosis signalling	10	4 (1%)	3 (0.06%)	3 (0.08%)

Percentages indicate proportion of proteins in a zone belonging to the specific functional class.

As we have shown that the zones closest to the centre are highly connected, it appears that a very important feature of the HFPIN is a highly connected signalling core, which may flexibly modulate responses to extracellular and intracellular stimuli via a large number of possible shortest paths to the rest of the network. It is likely that such signals emanate from within and between the innermost zones of HFPIN (zones 1 and 2), which are significantly enriched for signalling functions and where the largest number of possible of interactions occur amongst signalling proteins and with other important proteins. As almost all known diseases exhibitdysfunctional signalling networks [38], the extreme enrichment of zones 1 and 2 for signalling pathway functions makes the proteins in those belonging to that functional class potentially high priority novel drug target candidates.

### Essential, disease related, tumour suppressor, oncogenic and therapeutic target proteins are centrally located in human PINs

We extracted a list of human proteins that are likely to be essential based on the fact that knockouts of their orthologs in mice are annotated in the Mouse Genome Database as producing pre-, peri- and post-natal lethal phenotypes. These proteins comprise 43%, 21.7%, 10.7%, 9.3% and 9.6% of proteins in zones 1, zone 2, zone 3, zone 4 and zone 5 respectively of HFPIN and the differences between zones were confirmed to be statistically significant (P < 0.01).

We also determined in HFPIN the distribution of proteins annotated as being involved in at least one disease by the Disease Ontology Project [39]. Zones 1 to 6 were found to contain 159, 1184, 545, 85, 19 and 2 disease related proteins respectively. While zone 2 contains the largest number of disease gene products, the same pattern is displayed as for essential gene products, with 42.5%, 25.7%, 14.9%, 14.7%, 18.3% and 14.3% of proteins occurring in zones 1 to 6 respectively, being classified as such. The evidence strongly suggests that zones closest to the topological centre contain the largest numbers and proportions of important proteins, with zone 1 on the whole appearing to be most sensitive to aberrations.

We further tested this new hypothesis by determining the distribution of 49 known oncogenes and 62 suppressor genes [40]. Again, the majority of those proteins are located in zones 1 and 2 with zone 1 again having the highest proportion of its proteins belonging to those functional classes, with 4%, 0.6%, 0.02% and 0.1% of proteins in zones 1 to 4 being products of oncogenes, and 3.7%, 0.9%, 0.1% and 0.3%, respectively being tumour suppressors.

In order to determine whether the clear dominance of zone 1 and 2 proteins in essential roles and the diseases have potential implications for drug discovery, we assessed the zone distribution of 497 clinically approved human drug target proteins extracted from the Therapeutic Target Database [41]. The pattern of distribution is virtually identical to the aforementioned categories, where 497 proteins tested comprise 15.7%, 7.5%, 2.1% and 1.8% of proteins in zones 1 to 4, respectively and the differences between zones were again confirmed to be statistically significant (P < 0.01). As with the other protein functional classes tested, zone 1 contains proportionally the largest percentage of drug targets and approximately double that of zone 2, despite containing only 10% as many proteins. Further, more peripheral zones have comparatively much lesser numbers, and we therefore propose that proteins in zone 1 and 2 should be given priority in the search for novel drug target candidates and disease genes (Table 5).

**Table 5:** Distribution of essential, disease, drug target and classical cancer proteins in HFPIN zones

**Zone**	**Essential**	**Disease**	**Drug**	**Oncogenes**	**Tumour**
	**roles**	**associated**	**targets**		**suppressors**
1	161 (43%)	159 (42.5%)	59 (15.7%)	15 (4%)	14 (3.7%)
2	1002 (21.7%)	1184 (25.6%)	346 (7.5%)	32 (0.7%)	42 (0.9%)
3	392 (10.7%)	545 (14.9%)	77 (2.1%)	1 (0.02%)	4 (0.1%)
4	55 (9.3%)	85 (14.4%)	11 (1.8%)	1 (0.2%)	2 (0.3%)
5	10 (9.6%)	19 (18.2%)	3 (2.8%)	-	-
6	-	2 (14.2%)	1 (7.1%)	-	-

Surprisingly similar and statistically significant patterns were seen in the HSN (Table 6) and the distribution of all important cellular functions is essentially identical to the HFPIN.

**Table 6:** Distribution of essential, drug target and classical cancer proteins in HSN zones

**Zone**	**Essential**	**Drug**	**Oncogenes**	**Tumour**
	**roles**	**targets**		**suppressors**
1	157 (36.4%)	69 (16%)	12 (2.7%)	12 (2.7%)
2	815 (23.1%)	291 (8.2%)	33 (0.9%)	41 (1.1%)
3	268 (13.8%)	103 (5.3%)	4 (0.02%)	4 (0.2%)
4	29 (14%)	5 (2.4%)	-	-
5	4 (10.5%)	1 (2.6%)	-	1 (2.6%)

### The majority of consistently expressed proteins in cancers are located in zones closest to the centre of human PINs

Using absence/presence calls from the Gene Expression Barcode database, we identified genes which are consistently expressed in more than 99% of samples of a given cancer and mapped them onto the zones in which they occur in the human PINs. We found that these proteins are primarily located in zones closest to the centre of human PINs (Tables 7 and 8). Most of these are located in zone 2, followed by zone 3 and zone 1. In the periphery, the percentage gradually decreases up to zone 6, after which they are absent.

**Table 7:** HFPIN zone distribution of proteins consistently expressed in cancer samples

**Type of cancer**	**# proteins**	**Zone 1**	**Zone 2**	**Zone 3**	**Zone 4**	**Zone 5**	**Zone 6**
Breast	330	11 (3.3%)	189 (57.2%)	121 (36.6%)	9 (2.7%)	-	-
Cervical	711	26 (3.6%)	425 (59.7%)	230 (32.3%)	23 (3.2%)	7 (0.9%)	-
Endometrial	1515	57 (3.7%)	839 (55.3%)	514 (33.9%)	83 (5.4%)	20 (1.3%)	2 (0.1%)
Fallopian	1292	49 (3.7%)	715 (55.3%)	446 (34.5%)	67 (5.1%)	14 (1%)	1 (0.07%)
Glioblastoma	1046	38 (3.6%)	589 (56.3%)	368 (35.1%)	44 (4.2%)	6 (0.5%)	1 (0.9%)
Glioma	1180	40 (3.3%)	621 (57.7%)	440 (37.2%)	63 (5.3%)	13 (1.1%)	3 (0.2%)
Kidney	561	14 (2.4%)	331 (59%)	193 (34.4%)	23 (4%)	-	-
Liver	715	29 (4%)	402 (56.2%)	247 (34.5%)	33 (4.6%)	4 (0.5%)	-
Lung	532	19 (3.5%)	314 (59%)	175 (32.8%)	22 (4.1%)	2 (0.3%)	-
Ovarian	775	26 (3.3%)	432 (55.7%)	279 (36%)	32 (4.1%)	6 (0.7%)	-
Pancreatic	717	30 (4.1%)	411 (57.3%)	244 (34%)	28 (3.9%)	4 (0.5%)	-
Pituitary	1126	37 (3.2%)	591 (52.4%)	421 (37.3%)	61 (5.4%)	15 (1.3%)	1 (0.08%)
Rectal	1597	69 (4.3%)	861 (53.9%)	552 (34.5%)	90 (5.6%)	23 (1.4%)	2 (0.1%)
**Average**		**3.5%**	**56.5%**	**34.8%**	**4.4%**	**0.7%**	**0.1%**

**Table 8:** HSN distribution of proteins consistently expressed in cancer samples

**Type of cancer**	**# proteins**	**Zone 1**	**Zone 2**	**Zone 3**	**Zone 4**	**Zone 5**	**Zone 6**
Breast	236	12 (5%)	151 (63.9%)	70 (29.6%)	2 (0.8%)	1 (0.4%)	-
Cervical	533	42 (7.8%)	323 (60.6%)	157 (29.4%)	9 (1.6%)	1 (0.1%)	1 (0.1%)
Endometrial	1092	89 (8.1%)	647 (59.2%)	336 (30.7%)	17 (1.5%)	2 (0.1%)	1 (0.09%)
Fallopian	941	72 (7.6%)	563 (59.8%)	287 (30.4%)	16 (1.7%)	2 (0.2%)	1 (0.1%)
Glioblastoma	767	64 (8.3%)	471 (61.4%)	216 (28.1%)	13 (1.6%)	2 (0.2%)	1 (0.1%)
Glioma	824	35 (8%)	278 (64%)	114 (62.2%)	5 (1.1%)	1 (0.2%)	1 (0.2%)
Kidney	434	14 (2.4%)	331 (59%)	193 (34.4%)	23 (4%)	-	-
Liver	537	45 (8.3%)	328 (61%)	155 (28.8%)	7 (1.3%)	1 (0.1%)	1 (0.1%)
Lung	422	31 (7.3%)	260 (61.6%)	121 (28.6%)	8 (1.9%)	2 (0.4%)	-
Ovarian	557	39 (7%)	334 (59.9%)	174 (31.2%)	8 (1.4%)	1 (0.1%)	1 (0.1%)
Pancreatic	536	46 (8.5%)	332 (61.9%)	148 (27.6%)	8 (1.4%)	1 (0.1%)	1 (0.1%)
Pituitary	789	56 (7%)	458 (58%)	253 (32%)	19 (2.4%)	2 (0.2%)	1 (0.1%)
Rectal	1162	95 (8.1%)	677 (58.2%)	365 (31.4%)	21 (1.8%)	3 (0.2%)	1 (0.8%)
**Average**		**7.5%**	**60.6%**	**29.6%**	**1.5%**	**0.2%**	**0.1%**

### Proteins located in zones closest to the HFPIN’s centre dominate important and disease pathways

**Figure 1:**
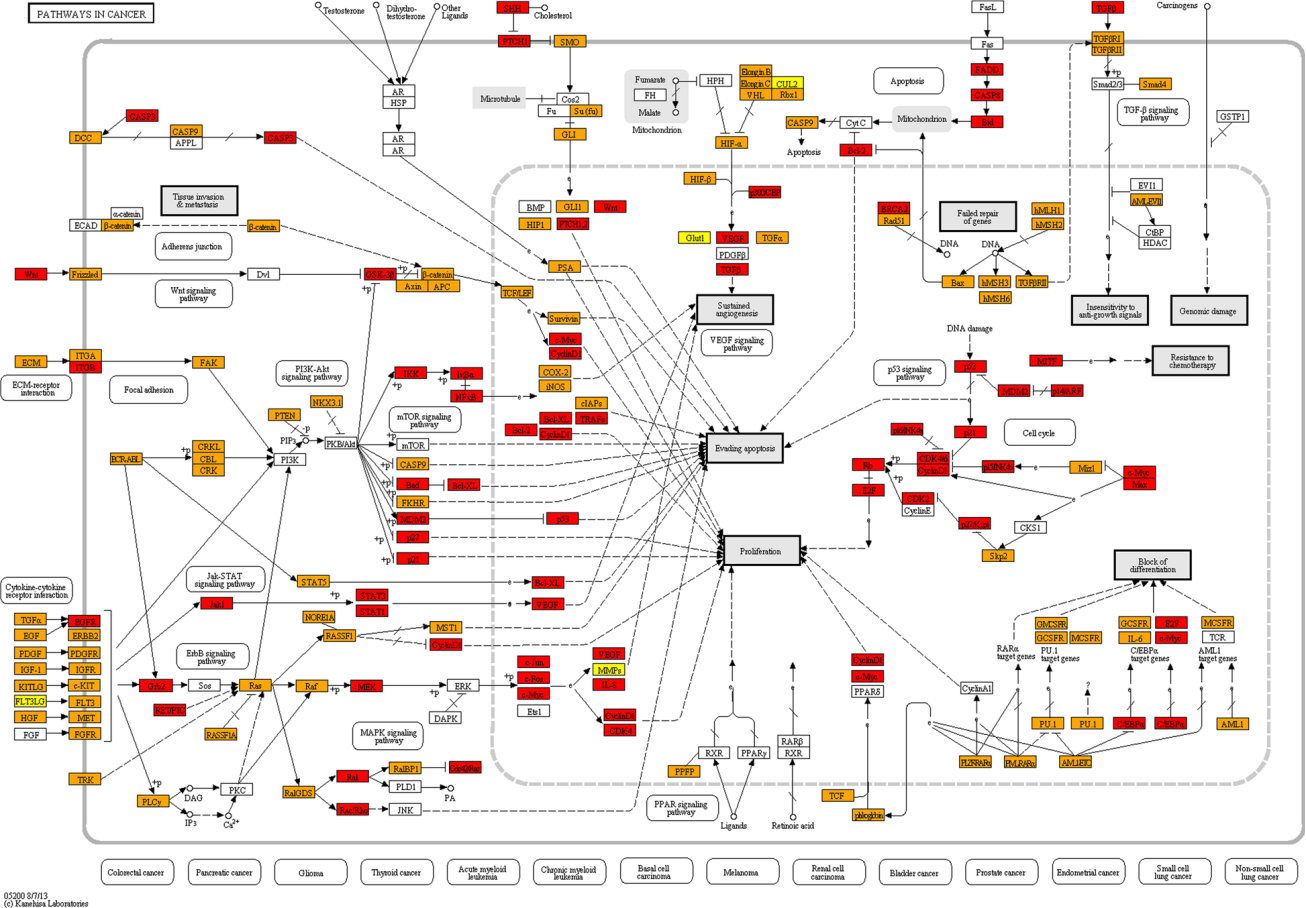
**KEGG ‘Pathways in Cancer’.** (Zone 1: red, zone 2: orange, zone 3: yellow).

**Figure 2:**
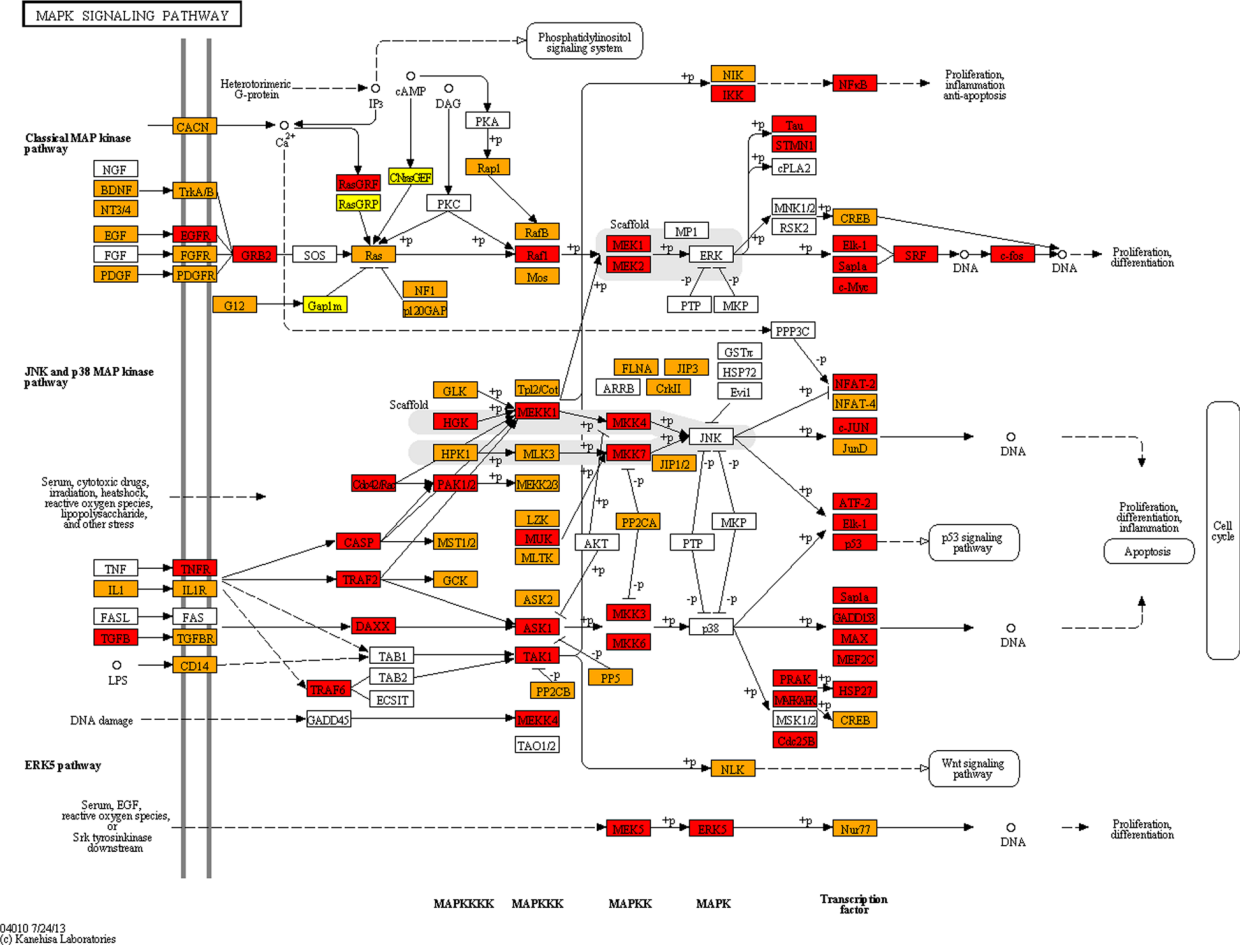
**KEGG MAPK signalling pathway.** (Zone 1: red, zone 2: orange, zone 3: yellow).

**Figure 3:**
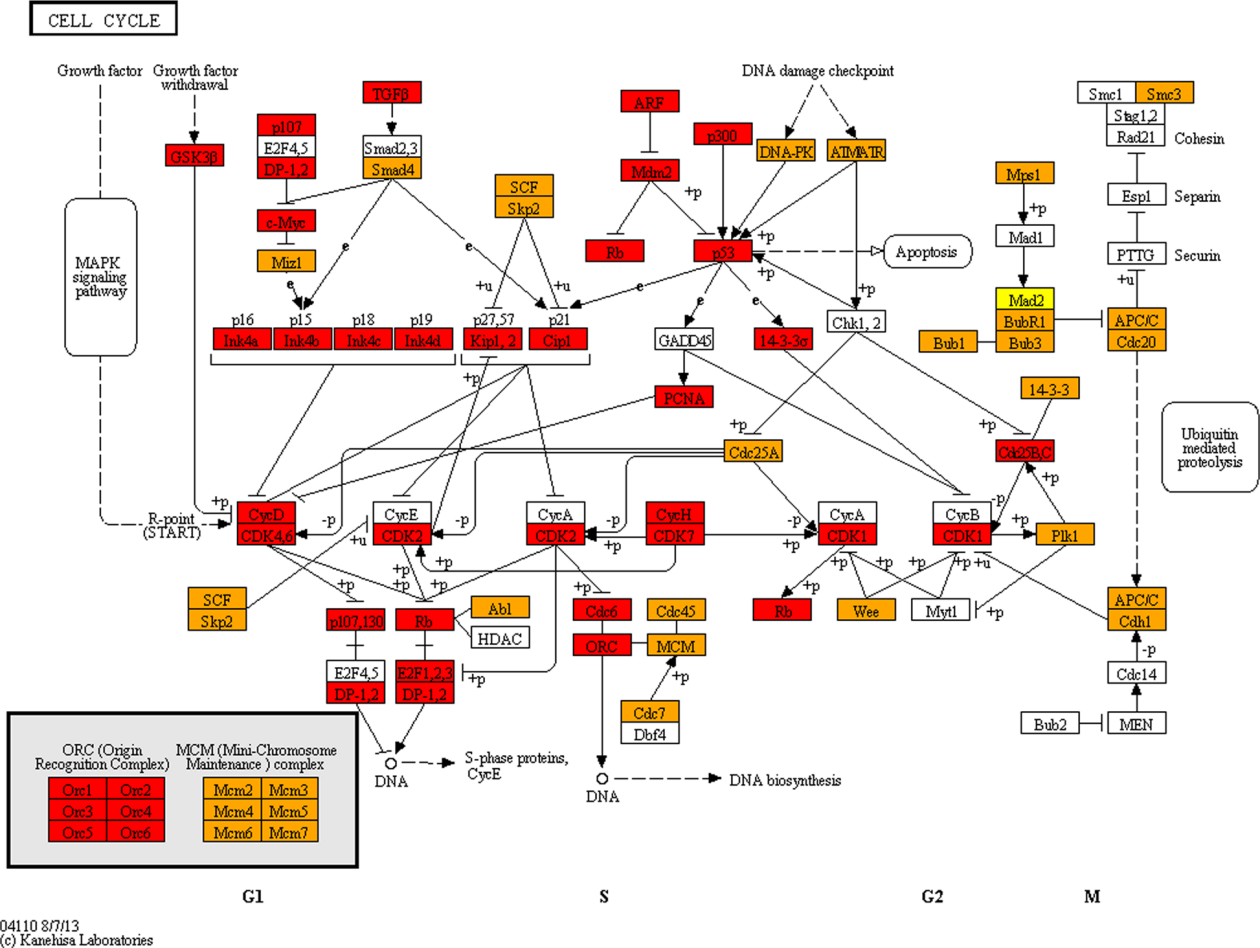
**KEGG Cell-cycle pathway.** (Zone 1: red, zone 2: orange, zone 3: yellow).

**Figure 4:**
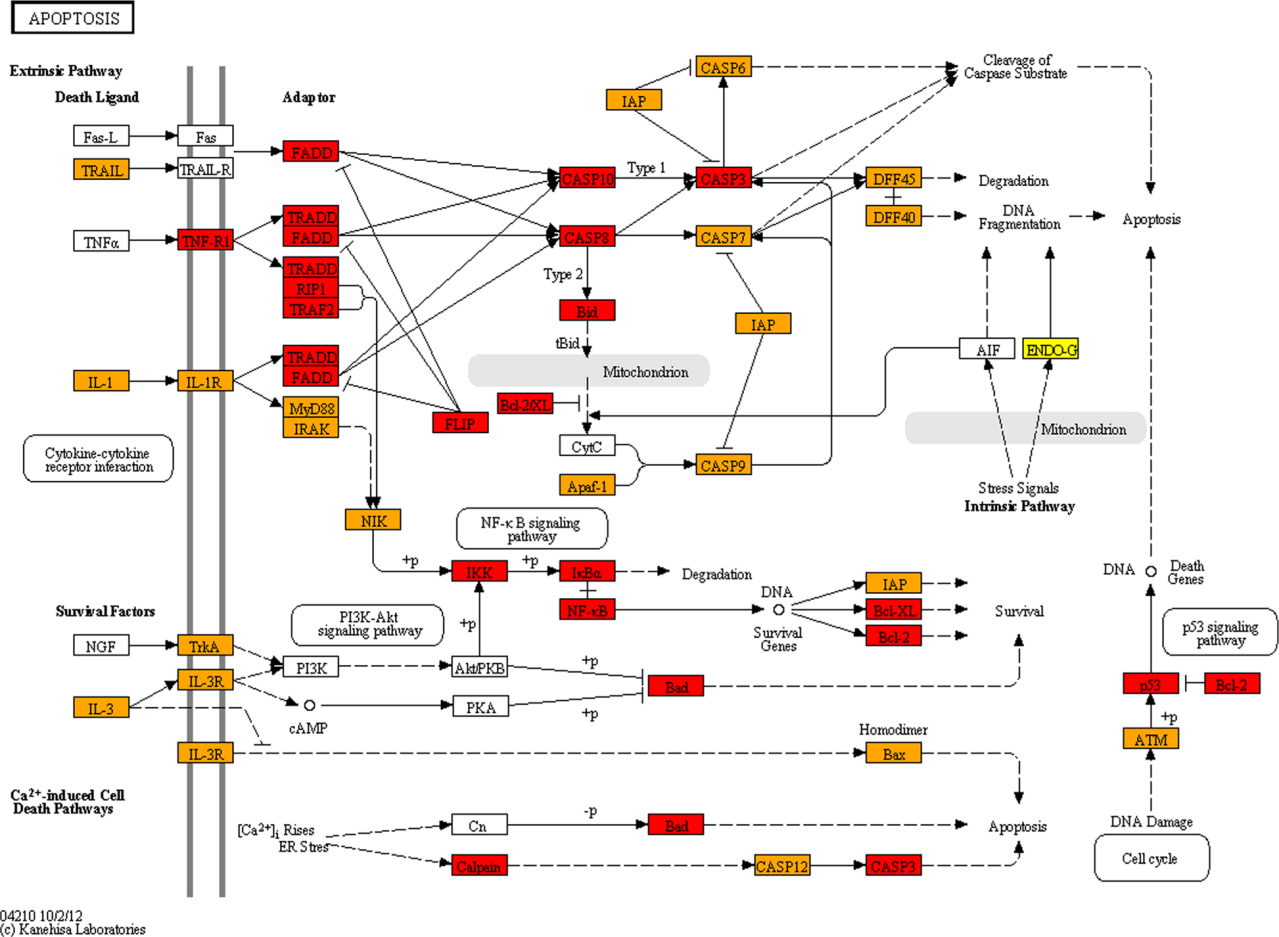
**KEGG Apoptosis pathway.** Zone 1: red, zone 2: orange.

In order to determine whether the enrichment for specific pathways in zones closest to the centre are in concordance with the proportions of proteins from those zones in the said pathways, we mapped HFPIN zone locations to proteins in KEGG pathways [42] using the KEGG Mapper facility (http://www.genome.jp/kegg/tool/map_pathway2.html). Strikingly, proteins from zone 1 of the HFPIN comprise a significant proportion of key pathways despite the fact that zones 2 and 3 contain approximately 10 times as many proteins. Furthermore, the vast majority of proteins involved in KEGG ‘pathways in cancer’, ‘MAPK-signalling’, ‘cell cycle’ and ‘apoptosis’ are from zones 1 and 2 (Figures 1, 2, 3 and 4, respectively). This dominance may be surprising given that the HFPIN represents less than half of known human proteins. Similar patterns are seen in the melanoma pathway and also in pathways for non-neoplastic diseases such as measles, inflammatory bowel disease and Alzheimer’s disease (Additional files 1, 2, 3 and 4, respectively). Also interesting is the observation that distinct sub-pathways are comprised of proteins from a specific zone. For example, in the melanoma pathway, all proteins involved in the cell cycle are located in zone 1. For the cell cycle pathway itself, all components of the origin recognition complex are from zone 1, while all in the mini-chromosome maintenance complex are from zone 2, which we propose adds further credence to our hypothesis that grouping proteins in PINs based on distances from the topological centre has biological significance. It is also apparent that proteins positioned closest to the topological centre of the HFPIN are involved in key roles within important cellular pathways as well as those leading to disease.

### Central zones of a combined network display increased bias for disease-linked and drug target proteins

We non-redundantly merged the HFPIN and the HSN into a combined network (CN) of 10573 nodes and 210689 interactions and modelled it as a metric space. Even though the core-periphery structure of the CN is similar to those of the original networks, the proportional sizes and connectivity of its zones 1 and 2 are significantly increased (Table 1). In line with our hypothesis on the cellular importance of central zones, the involvement of those zones in signal transduction and disease pathways is also increased (Table 9). Similar to HFPIN and HSN, zones closest to the centre of the CN also has the highest proportional representation of signalling functions (Table 10), essential, drug target and classical cancer proteins (Table 11) and proteins consistently expressed by specific cancers (Table 12). However, we propose that the drug discovery potential of our metric space model of the CN is further increased compared to the individual networks due to the higher number of proteins and hubs in its central zones. This was further supported when we mapped proteins onto KEGG pathways as before and found that CN zone 1 proteins feature even more prominently in key pathways than does the equivalent in HFPIN. Coloured pathway maps can be downloaded from ftp://ftp.sanbi.ac.za/junaid/bmc2/CN_colored_maps.zip.

**Table 9:** Functional specialization of CN zones defined by distance from the network centre

**Enriched Pathway**	**Zone 1**	**Zone 2**	**Zone 3**	**Zone 4**
Signal transduction	43*%*	21.6*%*	-	-
Immune system	32*%*	10.9*%*	5.4*%*	-
MAPK signalling	25.8*%*	2.3*%*	-	-
Pathway in cancer	20.7*%*	3.5*%*	-	-
Disease	22.6*%*	9.3*%*	5.4*%*	-
Hemostasis	15.9*%*	5.3*%*	-	-
Cell Cycle	5.4*%*	5.5*%*	3.1*%*	-
Gene expression	7.8*%*	8.3*%*	12.5*%*	-
Metabolism	13.1*%*	9.3*%*	8.8*%*	15.9*%*

Percentages indicate proportion of proteins in a zone belonging to the specific functional class.

**Table 10:** Distribution of signalling related functions in HFPIN and CN zones

	**HPFIN**			**CN**		
**Function**	***Zone 1***	***Zone 2***	***Zone 3***	***Zone 1***	***Zone 2***	***Zone 3***
Signalling	285 (76.2%)	2141 (46.4%)	760 (20.8%)	397 (73.2%)	2434 (40.4%)	640 (19%)
Positive regulation	75 (20%)	386 (8.3%)	83 (2.2%)	123 (22.6%)	393 (6.5%)	50 (1.4%)
Negative regulation	57 (15.2%)	321 (6.9%)	71 (1.9%)	82 (15.1%)	344 (5.7%)	54 (1.6%)
MAPK signalling	93 (24.8%)	258 (5.5%)	43 (1.1%)	139 (25.6%)	249 (4.1%)	20 (0.5%)

**Table 11:** Distribution of essential, drug target and classical cancer proteins in CN zones

**Zone**	**Essential**	**Disease**	**Drug**	**Oncogenes**	**Tumour**
	**genes**	**associated**	**targets**		**suppressors**
1	206 (38%)	234 (43.2%)	97 (17.8%)	17 (3.1%)	20 (3.6%)
2	1084 (18%)	1279 (21.3%)	365 (6%)	32 (0.5%)	42 (0.6%)
3	315 (9.3%)	415 (12.4%)	59 (1.7%)	2 (0.05%)	4 (0.1%)
4	28 (7.6%)	56 (15.3%)	3 (0.8%)	1 (0.2%)	1 (0.2%)
5	2 (3.2%)	9 (14.8%)	1 (1.6%)	-	-

**Table 12:** CN distribution of proteins consistently expressed in cancer samples

**Type of cancer**	**# proteins**	**Zone 1**	**Zone 2**	**Zone 3**	**Zone 4**	**Zone 5**
Breast	350	24 (6.8*%*)	224 (64*%*)	95 (27.1*%*)	7 (2*%*)	-
Cervical	760	43 (5.6*%*)	496 (65.2*%*)	203 (26.7*%*)	16 (2.1*%*)	2 (0.2*%*)
Endometrial	1644	91 (5.5*%*)	1007 (61.2*%*)	474 (28.8*%*)	61 (3.7*%*)	11 (0.6*%*)
Fallopian	1408	71 (5*%*)	869 (61.7*%*)	409 (29*%*)	51 (3.6*%*)	8 (0.5*%*)
Glioblastoma	1128	63 (5.5*%*)	719 (63.7*%*)	311 (27.5*%*)	30 (2.6*%*)	5 (0.4*%*)
Glioma	1270	67 (5.2*%*)	765 (60.2*%*)	380 (29.9*%*)	48 (3.7*%*)	10 (0.7*%*)
Kidney	593	44 (7.4*%*)	389 (65.5*%*)	150 (25.2*%*)	10 (1.6*%*)	-
Liver	769	51 (6.6*%*)	475 (61.7*%*)	221 (28.9*%*)	21 (2.7*%*)	1 (0.1*%*)
Lung	571	39 (6.8*%*)	369 (64.6*%*)	153 (26.7*%*)	9 (1.5*%*)	1 (0.1*%*)
Ovarian	823	37 (4.4*%*)	524 (63.6*%*)	236 (28.6*%*)	23 (2.7*%*)	3 (0.3*%*)
Pancreatic	771	44 (5.7*%*)	483 (62.6*%*)	223 (28.9*%*)	21 (2.7*%*)	-
Pituitary	1228	60 (4.8*%*)	738 (60*%*)	373 (30.3*%*)	47 (3.8*%*)	10 (0.8*%*)
Rectal	1753	96 (5.4*%*)	1061 (60.5*%*)	515 (29.3*%*)	70 (3.9*%*)	11 (0.6*%*)
**Average**		**5.7%**	**62.7%**	**28.2%**	**2.8%**	**0.3%**

## Conclusion

Our over-representation analysis on zones depending on the distance from the centre of network has shown that innermost zones of the human PINs are enriched for critically important proteins are functionally specialized. In addition, the majority of known disease-associated and drug target proteins are located in the first two zones. We therefore posit that other proteins in these centralpositions have similar importance, with zone 1 being particularly enriched for signal transduction proteins, an important class of therapeutic targets. We therefore propose that when considered holistically, central proteins having similar functions to existing drug targets are also potential targets for novel therapeutics. Similarly, based on our observation that disease pathways are dominated by central proteins, we propose that genes shortlisted in genome scale disease studies can be further prioritized based on whether their protein products occupy central positions in the human PINs. Further, the increased concentration of known therapeutic targets in zone 1 of the combined network compared to the other networks, along with its increase in the total number of proteins and average number of interactions, indicates that adding the information from the highly curated human signalling network to human PPI networks may significantly improve their utility in disease gene and drug target discovery.

## Methods

### Zones data sources

We consider zones of the human PINs as described previously [32]. We also non-redundantly merged the HFPIN and the HSN into a combined network. Proteins from all three networks classified into zones relative to the centre can be downloaded from ftp://ftp.sanbi.ac.za/junaid/bmc2/Zones_in_PPI_networks.zip.

### Functional enrichment analysis

In order to determine whether zones of the human PINs have biological significance, we divided proteins into subsets based on their distance from the true topological centre. Protein sets representing each zone was then subjected to a pathway over-representation analysis in order to determine whether the zones were specialized for specific functions. The Comparative Toxigenomics Database’s Gene Set Enricher web service (http://ctdbase.org/tools/enricher.go) was used to perform the enrichment analysis and a Bonferroni corrected p-value of 0.01 was chosen as a statistical significance cutoff. Lastly, when such enrichment was observed, we calculated the proportion of proteins involved in each enriched pathway as a way to assess whether any zones display functional specialization.

### Cancer gene expression data sources

We consider gene expression absence/presence calls from the following cancers types: breast, lung, kidney, pancreas, liver, cervix, ovary, glioblastoma, pituitary, glioma, fallopian, endometrium and rectum, which was downloaded from Gene Expression Barcode database (http://barcode.luhs.org/index.php?page=genesexp). Genes expressed in at least 99% of samples of a cancer of interest based on the Human HGU133 platform were downloaded. Gene expression was used as a proxy for protein expression and was mapped onto the PINs of interest in order to identify the zones in which gene product is located in.

### Testing the difference between proportions

We perform a z-test for the difference between two population proportions *p*_1_ and *p*_2_. We identify the null and alternative hypotheses and we specify the level of significance to be P < 0.01. After that we determine the critical value(s) from the statistic table. Finally we find the standardized test statistic as showing below.

### Statistical significance of the proportional analysis of pathway representation of zones

To test differences between proportions among zones, we need a statistical comparison of observed differences. A two-sample z-test for the difference between proportions for the top statistically enriched REACTOME pathways among zones was conducted. We defined the null hypothesis *H*_0_ to be: classification proportions of zones in the periphery in human PINs are as have high proportion significance as zones closest to the centre, i.e the accuracy of the sensing function are in zones closest to the centre and the accuracy of metabolic function are in zones in the periphery. If the *P*<0.01, we rejected *H*_0_ and concluded that the proportions support our claim that zones closest to the centre have high proportion significance than the zones in the periphery. In the other words, we have enough evidence at the 1% level to conclude that zones closest to the centre have high proportion significance than the zones in the periphery.

## Competing interests

The authors declare that they have no competing interests.

## Authors’ contributions

EF implemented the algorithms, performed the analyses and drafted the original manuscript. ECM proposed the concept of analyzing PINs as a metric spaces and oversaw the topological and statistical analyses. JG designed and oversaw and assisted in the functional evaluation tests and the biological interpretation of the results. ECM and JG supervised the study and edited the manuscript. All authors have read and approved the final manuscript.

## Supplementary Material

Additional file 1 **KEGG melanoma pathway.** Zone 1: red, zone 2: orange. Click here for file

Additional file 2 **KEGG measles pathway.** (Zone 1: red, zone 2: orange, zone 3: yellow), zone 4: green. Click here for file

Additional file 3 **KEGG inflammatory bowel disease pathway.** (Zone 1: red, zone 2: orange, zone 3: yellow, zone 4: green). Click here for file

Additional file 4 **disease pathway.** (Zone 1: red, zone 2: orange, zone 3: yellow, zone 4: green). Click here for file
