# Supplementary figures and images for: Modelling human protein interaction networks as metric spaces has potential in disease research and drug target discovery
Source: BMC Syst Biol. 2014 Jun 14;8:68. doi: 10.1186/1752-0509-8-68 (PMC4088370; doi:10.1186/1752-0509-8-68)

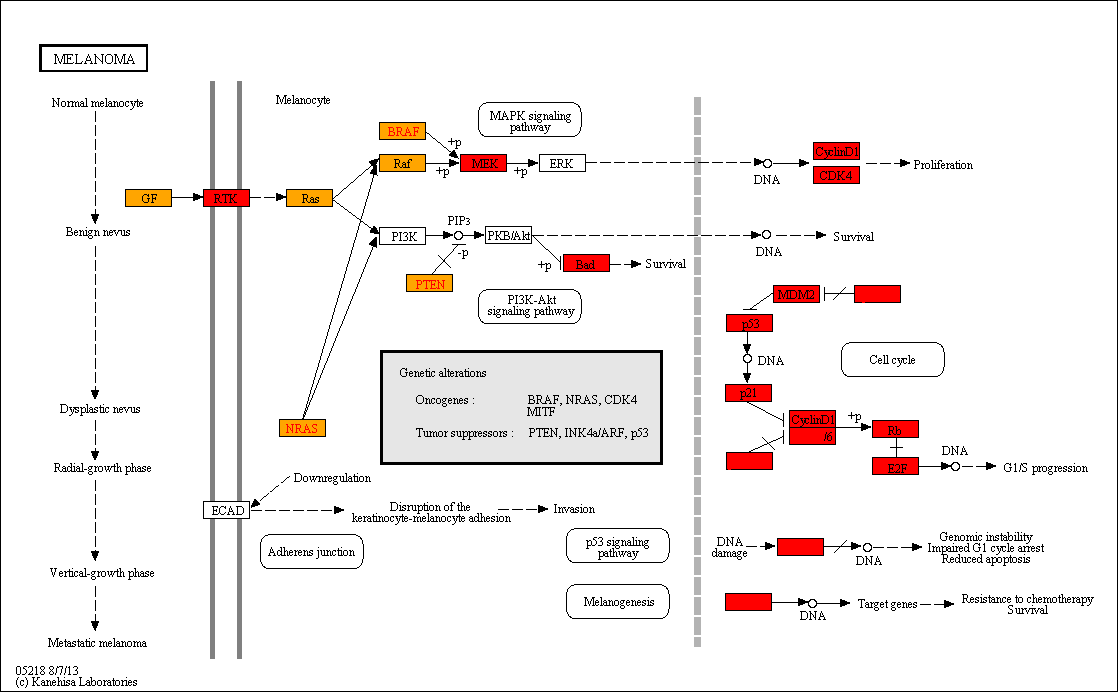

Supplement: Additional file 1 — KEGG melanoma pathway. Zone 1: red, zone 2: orange. [file 1752-0509-8-68-S1.png]

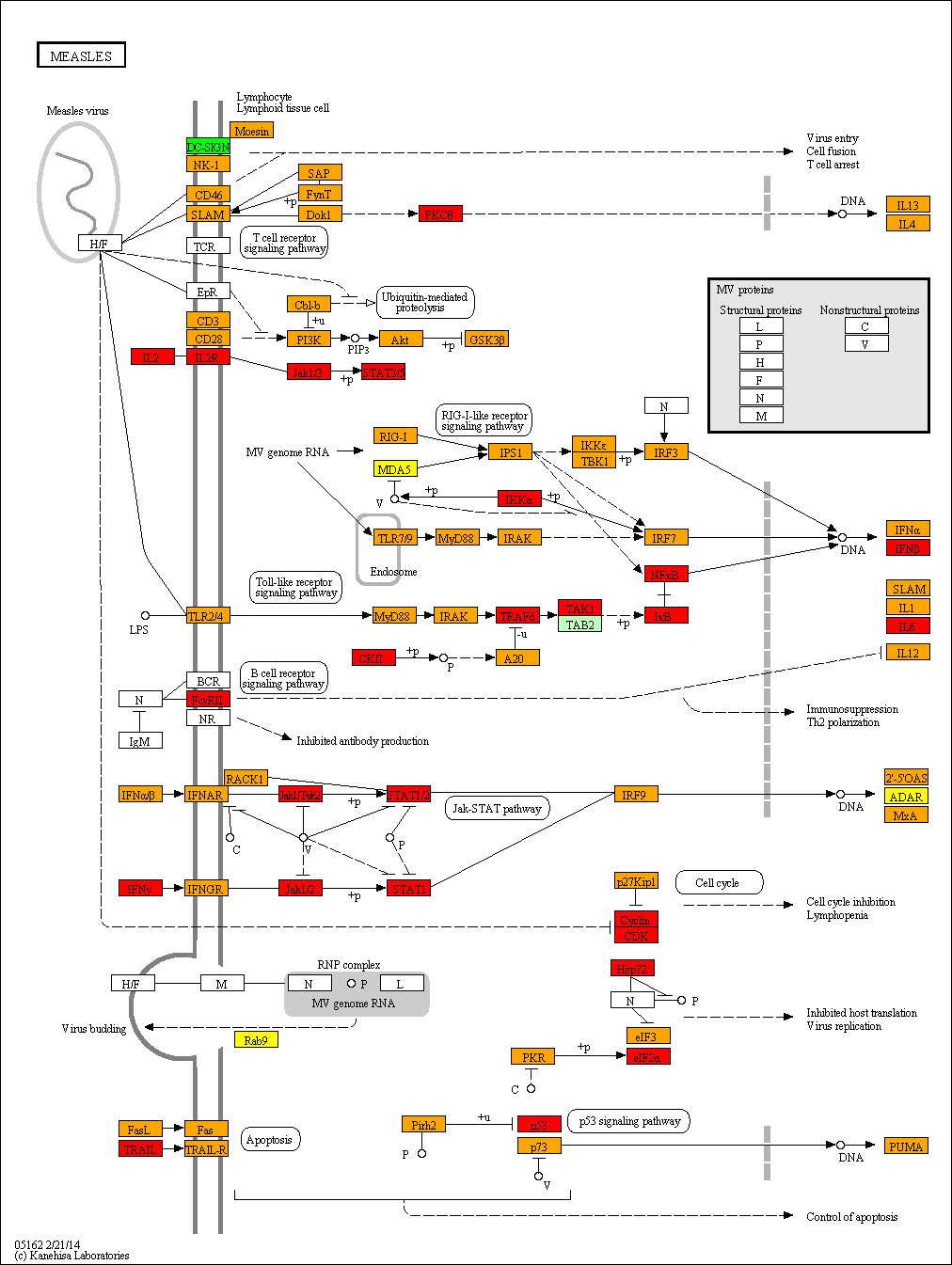

Supplement: Additional file 2 — KEGG measles pathway. (Zone 1: red, zone 2: orange, zone 3: yellow), zone 4: green. [file 1752-0509-8-68-S2.png]

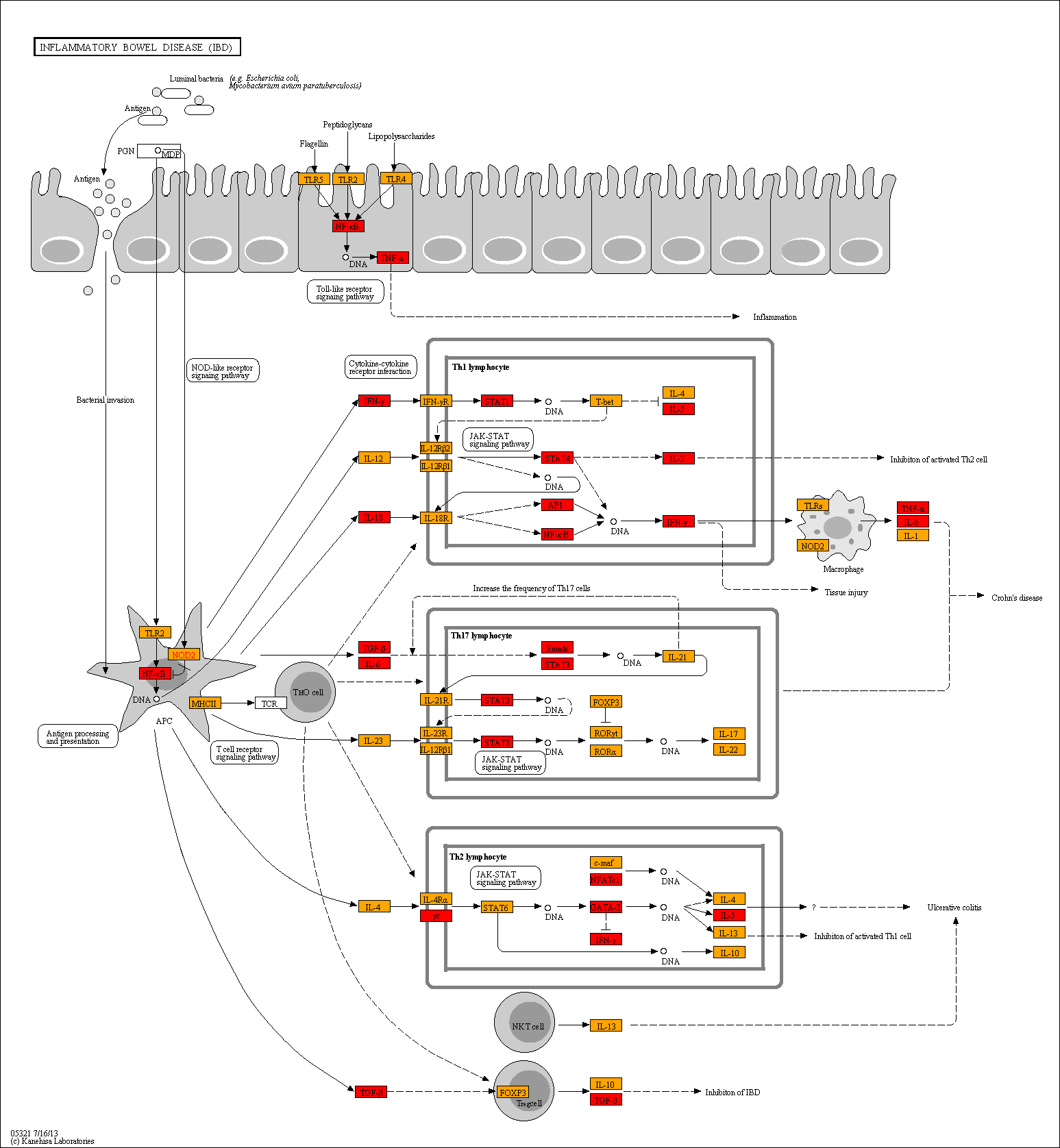

Supplement: Additional file 3 — KEGG inflammatory bowel disease pathway. (Zone 1: red, zone 2: orange, zone 3: yellow, zone 4: green). [file 1752-0509-8-68-S3.png]

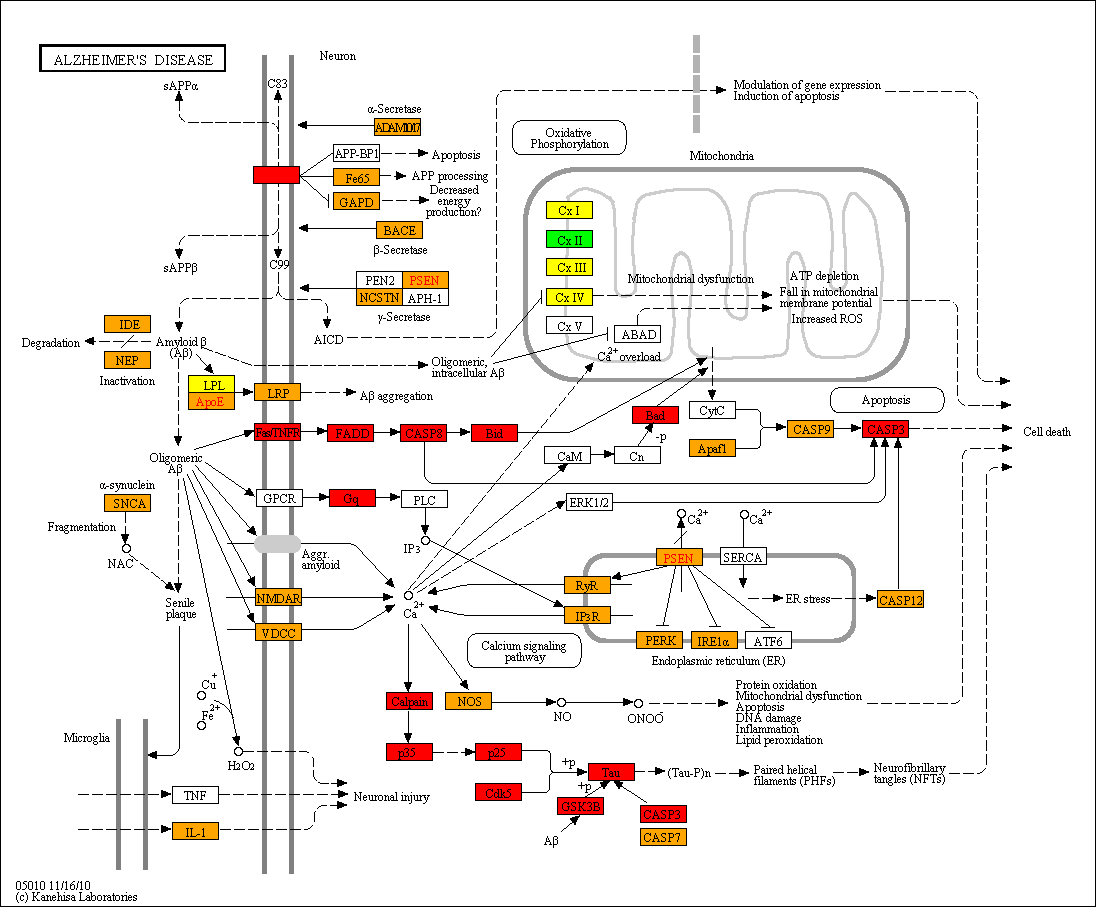

Supplement: Additional file 4 — disease pathway. (Zone 1: red, zone 2: orange, zone 3: yellow, zone 4: green). [file 1752-0509-8-68-S4.png]
